# Adiponectin in Relation to Coronary Plaque Characteristics on Radiofrequency Intravascular Ultrasound and Cardiovascular Outcome

**DOI:** 10.5935/abc.20180173

**Published:** 2018-09

**Authors:** José Mariani Junior

**Affiliations:** Hospital Israelita Albert Einstein, São Paulo, SP - Brazil; Instituto do Coração - HCFMUSP, São Paulo, SP - Brazil; Santa Casa de São Paulo, São Paulo, SP - Brazil

**Keywords:** Inflammation/physiopathology, Atherosclerosis, Biomarkers/blood, Plaque, Atherosclerotic/diagnostic imaging, Plaque, Atherosclerotic/genetics, Ultrasonography, Interventional, Adiponectin

Inflammation is a complex and necessary component of the response to biological, chemical, or physical stimuli, and the cellular and molecular events that initiate and regulate the interactions between the various players in the inflammatory process of the atherosclerotic lesions remain a source of ongoing investigation.^1^

The European Collaborative Project on Inflammation and Vascular Wall Remodeling in Atherosclerosis - Intravascular Ultrasound (ATHEROREMO-IVUS) study aimed to investigate the relations of genetic profile and novel circulating and inflammatory biomarkers with coronary plaque phenotype and vulnerability as determined by intravascular ultrasound (IVUS).^2^ Results from this trial have been helping us to improve our knowledge on the role of genetic profile and circulating inflammatory biomarkers in relation to the development of atherosclerosis and vulnerable plaques.

As an endocrine organ, adipose tissue is recognized as a rich source of pro-inflammatory mediators that may directly contribute to vascular injury, insulin resistance, and atherogenesis.^3^ Therefore, this kind of tissue may modulate inflammatory response by releasing a wide range of mediators, known as adipocytokines. Adiponectin, a kind of adipocytokines, has antiatherogenic and anti-inflammatory properties and acts as a factor increasing insulin sensitivity, and its protective effect may result from its ability to suppress production of proinflammatory cytokines.^3^ Due to the complex balance between pro- and anti-inflammatory activity, their pathophysiological and prognostic role in cardiovascular diseases still remains debated.^4^

Adiponectin, tested in this trial, was presented herein by Marino BCA et al.^5^ Although the median of its serum value in the complete cohort is not associated to the composition of the atherosclerotic plaque nor to the plaque burden assessed by Virtual Histology IVUS (VH-IVUS), it may be considered as an independent variable of death in this sample. Differently, in the sub-group of patients with stable symptoms, adiponectin median value was associated to a thin-cap fibroatheroma identified by VH-IVUS, but not to death.

These discrepancies reveal the difficulty in this elegant way in attempting to identify any biomarker for the recognition of patients with vulnerable atherosclerotic plaques and therefore at high risk for hard outcomes such as death. But it also shows that this is a long way, but one that must be pursued and validated, and then, try to find out what else, besides the measures already known, can be added to this so special group of patients and with a such high risk.
